# Barriers and Facilitators to Accessing and Using Maternal Healthcare Services by Women Living in Rural Bangladesh: A Theory-Guided Narrative Literature Review

**DOI:** 10.3389/phrs.2025.1608157

**Published:** 2025-12-29

**Authors:** Sanjoy Kumar Chanda, Gretl A. McHugh, Maria Horne

**Affiliations:** 1 Sociology Discipline, Social Science School, Khulna University, Khulna, Bangladesh; 2 School of Healthcare, Faculty of Medicine and Health, University of Leeds, Leeds, United Kingdom

**Keywords:** barriers and facilitators, access, maternal healthcare, women, rural Bangladesh

## Abstract

**Objective:**

To identify and synthesize the barriers and facilitators to accessing and using maternal healthcare (MHC) services by women living in rural Bangladesh.

**Methods:**

A structured literature search was conducted using six databases in 2024. Studies were synthesized using a thematic approach, underpinned by the Social-Ecological Model.

**Results:**

Searches returned 3,619 studies, of which 37 were included in this review. Findings related to barriers and facilitators were categorized into four themes: individual, family, social and community, and organizational levels. Key barriers to accessing and using MHC services included illiteracy, lack of family support, cultural taboo to pregnancy disclosure, distance to health facilities, and lack of quality services. Key facilitators to accessing and using MHC services were higher literacy levels, family support, NGO support and mass media exposure, and free healthcare services.

**Conclusion:**

Based on the findings of the review, to improve Bangladeshi women’s access to and use of MHC services, improvements in women’s literacy levels, village roads, family support, and service-related skills of healthcare providers are necessary.

## Introduction

The United Nations Sustainable Development Goal 3 (SDG3) aims to ensure healthy lives and promote wellbeing for all by 2030 [1]. Strengthening maternal healthcare (MHC) services for women, including antenatal care (ANC), childbirth, and postnatal care (PNC), are considered essential components to achieving the SDG3 targets [2].

Bangladesh made notable advancements in maternal health through MHC services in response to the call of the Millennium Development Goals, and is currently working toward the newly agreed SDGs to be achieved by 2030 [3–5]. The newly agreed SDG3 related to maternal health includes reducing the maternal mortality rate (MMR) to 70 or less per 100,000 live births, ensuring universal access to sexual and reproductive healthcare services, achieving universal health coverage, etc. [1]. However, the Sustainable Development Report (SDR) 2024, with an aim to provide updated information to track performance on the SDGs, shows a moderate improvement in the MHC situation in Bangladesh [6]. The SDR Report classifies Bangladesh inside the moderate progress category, as its current SDG performance score is 64.3, exceeding the 50% threshold of the required growth rate but falling short of the 70% necessary to attain the SDG by 2030 [6]. Therefore, Bangladesh is only partially on track to meet SDG3-related targets. Moreover, several socioeconomic issues, such as poverty, lack of healthcare infrastructure, etc., remain challenges in terms of progress toward meeting the SDG3 targets [6]. Access to and utilization of MHC services remains difficult for some population groups within Bangladesh, for example, married women who reside in remote rural regions of Bangladesh [5]. Women residing in rural areas in Bangladesh account for 35% of the female population and are less likely than urban women (57%) to have made the World Health Organization (WHO) recommended four or more ANC visits in 2022 [7]. Although the target of the Government of Bangladesh was to reach 50% coverage of four or more ANC visits by 2023, the percentage of women living in rural Bangladesh attending four or more ANC visits declined from 43% in 2017/18%–35% in 2022 [3, 7].

In relation to facility-based childbirth, whilst more than half (68%) of urban-dwelling childbirths in Bangladesh were attended by qualified doctors, nurses or other healthcare staff in Bangladesh, this rate is almost half for mothers residing in rural Bangladesh (51%) [7]. Moreover, Bangladesh is one of the largest contributors to the global burden of maternal death, with an estimated 123 MMR per 100,000 in 2020 compared to the UK, where the MMR rate is only 10 per 100,000 during the same period [5, 8].

Maternal deaths in Bangladesh are reported in the first 2 days after childbirth, and as a result, prompt PNC is important for the mother to identify and treat any complications arising from childbirth. The percentage of non-institutional childbirth that occurs predominantly in rural areas, for which the mother received PNC from a medically trained provider within 2 days of childbirth, was reported only to be 13% in 2022 [7].

Several challenges to access and use of MHC services include inadequate budget allocation resulting in reduced facilities, increased out-of-pocket costs [9, 10], lack of information and education about services, superstitions, and fear of losing family status [10], which negatively impact the availability and quality of maternity services, especially in rural Bangladesh. In addition, healthcare services-related challenges exist and include a lack of quality services, insufficient drug supplies [10, 11], and a shortage and absenteeism of physicians, as they prefer not to go to rural areas because of a lack of training opportunities, huge work pressure, and a preference to treat the urban educated class [9, 12, 13].

Therefore, to promote the overall health and wellbeing of Bangladeshi women and to make strides in achieving the SDG3 goal [1], it is important to understand the barriers and facilitators experienced by women in accessing and using MHC services in rural Bangladesh. To date, three reviews [14–16] have been undertaken related to healthcare services provision in Bangladesh. Two of the identified reviews have focused on noncommunicable diseases [15] and adolescents’ MHC services [16]. Only one review was relevant to the women’s MHC services [14], which was undertaken in 2011, and thus somewhat outdated. The review undertaken in 2011 reported that several barriers, including long distance between households and healthcare centers, poor income, unfriendly attitudes of healthcare providers, and the sex of providers, made women’s access and use of MHC services in rural Bangladesh acute [14]. The review is expected to be outdated, as the Government of Bangladesh has been actively engaged in the health sector since 2015, focusing on SDGs and initiating the digitalization process, resulting in numerous enhancements in MHC services. Furthermore, during the past decade since 2011, numerous research papers have been published that have not undergone review. Therefore, this narrative literature review intends to update the research literature in the MHC area using a theory-based approach.

**FIGURE 1:**
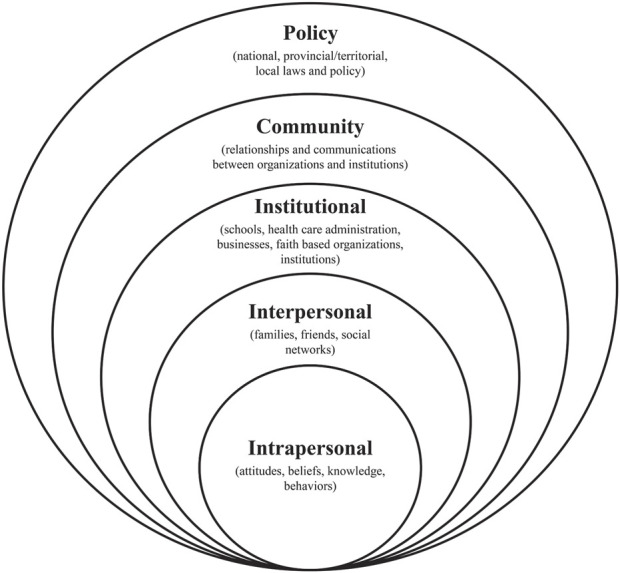
The Social-Ecological Model [17] (Literature review, Bangladesh, 2018–2024).

The Social-Ecological Model (SEM) can be used to understand healthcare access and utilization of individuals [5, 17], as it considers the dynamic interactions between individuals and their environments as determinants of health-related behavior [18]. The SEM shows that individuals’ healthcare access behavior is shaped through five hierarchical levels: intrapersonal, interpersonal, institutional, community, and policy levels [17] (Figure 1).

Therefore, this review aimed to identify and critically synthesize published studies examining barriers and facilitators to accessing and utilizing MHC services by women living in rural Bangladesh using the SEM.

## Methods

A narrative literature review [19] was undertaken using the Preferred Reporting Items for Systematic Reviews and Meta-Analyses (PRISMA) guidelines [20] as the step-by-step process for reporting on the review. A narrative literature review [19] was considered the most appropriate design to gain a better understanding of the barriers and facilitators to MHC services from the heterogeneous nature of the literature rather than summarizing results in response to a narrow research question, undertaken for a conventional systematic review [21]. To guide the review process, the SEM framework was adopted to provide a more holistic understanding of access to and utilization of MHC services by women residing in rural Bangladesh.

### Search Strategies

A systematic search was conducted with six electronic databases, including MEDLINE, PubMed, Web of Science, Scopus, ProQuest Sociological Abstract, and ProQuest Dissertation and Theses, to identify studies for inclusion in the review. For the MEDLINE database search, the Ovid interface was used, and for the PubMed database search, as PubMed includes all MEDLINE records, only resources apart from MEDLINE (e.g., PubMed Central articles) were used. All databases encompass published manuscripts in health and biomedical sciences, social sciences, public health, and nursing, offering relevant insights into the specific topic of interest [5]. The search with electronic databases was first undertaken in November 2019 and then updated in June 2024. To identify additional literature, first conducted in 2019 and then repeated in June 2024, Google Scholar, Google, and Leeds University Library were accessed using keywords (Table 1). The search included medical subject headings (MeSH) and text words for barriers and facilitators, maternal healthcare access, and maternal healthcare utilization. The Population, Intervention, Comparison, and Outcome (PICO) model (see an example in Table 1) was used to develop the search terms from the research question: What are the barriers and facilitators to accessing and using MHC services by rural Bangladeshi women? An example of search terms in MEDLINE (Ovid) has been included in Supplementary Material S1. Study titles and abstracts were screened first, and then full-text versions of potential citations were retrieved for detailed examination based on the eligibility criteria below.

**TABLE 1:** Identification of search words for barriers and facilitators to accessing and using maternal healthcare services by women in Bangladesh based on the Population, Intervention, Comparison and Outcome model (Literature review, Bangladesh, 2018–2024).

Population/problem	Intervention	Comparison	Outcome 1	Outcome 2
Women and Bangladesh	Barriers and facilitators	No	Healthcare access	Healthcare utilization
Women or female And Bangladesh* or India or Pakistan or Nepal or Bhutan or south asia or developing countr* or low-and middle-income countr*	Barrier* or discriminat* or difficult* or challenge* AND facilitator* or motivat* or enabler*	​	Healthcare access* or healthcare access* or community healthcare* or antenatal care* or postnatal care* or primary healthcare* or medical care*	Healthcare* utili#ation or healthcare utili#ation or healthcare application* or healthcare employment* or healthcare practice* or healthcare operation*

### Eligibility Criteria

Studies were included in the review if they: (i) incorporated access to and use of MHC services by women aged 16–49; (ii) were full-type peer-reviewed studies published in the English language; (iii) were conducted in Bangladesh; and (iv) were published after 2000. This review examined changes in MHC services in Bangladesh over the past two decades as the nation’s healthcare system has undergone changes during that time [5]. For example, in 1998, community clinics were built to provide MHC services to women residing in rural Bangladesh [22]. As a result, studies pertaining to these services were not published before 2000.

Studies were excluded when they included texts not available in full and primary data, such as conference abstracts, commentaries, editorials, and opinion papers.

### Study Selection

**FIGURE 2:**
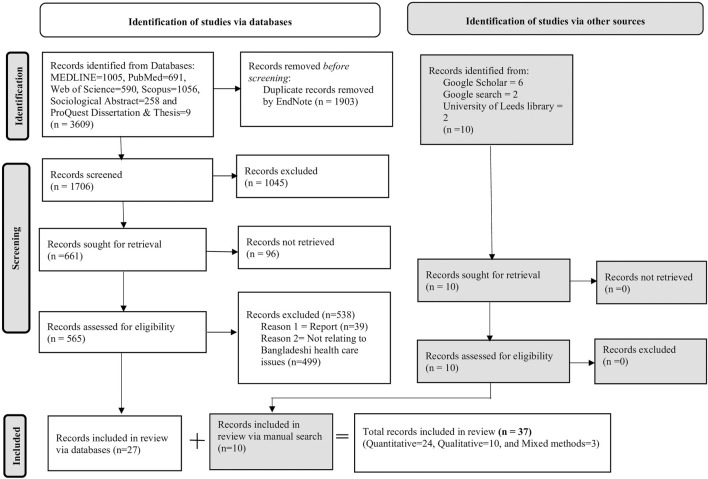
Flowchart of searches of databases and screening process based on the Preferred Reporting Items for Systematic Reviews and Meta-Analyses (Adapted from Page, et al. [20]) (Literature review, Bangladesh, 2018–2024).

A total of 3,619 publications were identified from electronic databases and manual searches. 1903 publications from the electronic databases were removed because of duplication, and the remaining 1706 abstracts were screened. Duplicate detection of studies was executed using EndNote software and a manual process where each individual study was checked. Of these, 1,045 publications were excluded based on the exclusion criteria. The full texts of the remaining 565 studies were examined in detail, and 538 studies were further excluded. Therefore, 27 studies were included from the database search. Additionally, 10 studies were identified from a manual search of the reference lists of the studies included, and they were all included after assessment. Finally, 37 studies were considered eligible and included in this review. A PRISMA flowchart was used to enhance the transparency and reporting of the literature search and selection process, as summarized in Figure 2.

### Assessing Quality of the Included Studies

The selected studies were assessed to determine their level of quality. As the review encompassed multiple study designs, three different appraisal checklists were used to assess qualitative, quantitative, and mixed methods (MM) studies (Supplementary Material S2), as recommended in the literature [23] and found in practice [24]. For this assessment process, cohort and randomized control trial (RCT) studies were assessed using the Critical Appraisal Skills Programme (CASP) checklists [25, 26], and cross-sectional quantitative studies using the critical appraisal checklist for cross-sectional studies that was adapted from Combie [27]. Additionally, the CASP checklist [28] was used to appraise qualitative studies, and the mixed methods appraisal tool (MMAT) version 2018 [29] for MM studies. Appraisals included a minimum of 10 and a maximum of 17 questions with possible answers of “Yes,” “No,” or “Can’t tell.” Each question contains one score.

To classify the quality of studies included in this review, a scoring procedure used in another review [24] was adopted for all the appraisals: (i) the individual score for each study was calculated based on the appraisal questions in each row of the Table (Supplementary Material S2); (ii) the score achieved was divided by the total score, and then converted into a percentage; and (iii) finally, three categories based on the percentage and range were made to classify the quality of the studies: 80%–100% = high, 60%–79% = medium, and <60% = low. Descriptive responses were not counted for scoring, and explanations were provided for each question.

### Data Synthesis

Thematic synthesis [30] was used for data synthesis as this review included heterogeneous study designs, and is recommended by the literature [30, 31]. There are trends in the use of thematic synthesis for narrative review in healthcare research where there is heterogeneity in studies [32, 33]. Thematic synthesis is considered an adaptation of thematic analysis to synthesize the research studies and offers a range of established methods and techniques for the identification and development of analytic themes in first-hand data [30]. Thematic synthesis [30] based on the SEM [17] was undertaken. Based on the SEM levels of influence, the extracted data were inductively coded, and themes and subthemes were then identified (Supplementary Material S3).

The characteristics and key findings were extracted and tabulated according to the Ma et al. [18] guidelines. The main characteristics include: (i) author(s) and year of publication, (ii) region where the study was conducted, (iii) aims of the study, (iv) study design, (v) sampling method and participants, (vi) types of healthcare services, and (vii) main findings, including barriers and facilitators. Initially, the studies were grouped and summarized separately on the basis of quantitative or qualitative design. Likewise, data obtained from the quantitative and qualitative components of the MM studies were included in the relevant group. The characteristics and key findings of these studies are summarized and categorized in Table 2.

**TABLE 2:** Characteristics of the included studies (Literature review, Bangladesh, 2018–2024).

Author and year	Region	Aim	Study design	Sampling method and participant	Type of healthcare service	Key findings	
						Barriers	Facilitators
Huda et al. [34]	Rural-urban	To examine the horizontal inequity in access to facility delivery in Nepal, Pakistan and Bangladesh, and identify the different need factors as well as other social determinants that can potentially explain such inequity in the use of the facility delivery services in these countries	Quantitative (survey)	Not reported and Women	MHC-childbirth	• Poverty, low level of literacy of both women and their husbands and higher parity were significant barriers to women accessing care	• Belonging in wealthy families, having higher education of both women and their husbands, living in urban area emerged as the most important factors in accessing the facility-based childbirth for women
Islam and masud [35]	Rural-urban	Examined the levels and determinants of frequency and contents of ANC visits in Bangladesh	Quantitative (survey)	Stratified-cluster sampling design and Women	MHC-ANC	• High birth order, residing in rural area were barriers for women in seeking MHC services	• Women’s high level of education, low parity, planned pregnancies, and media exposure were related to their ANC visits
Rahman et al. [36]	Rural-urban	To examine the association of reported complications around delivery and socio-demographic, healthcare and spatial characteristics of mothers with CS, using data from the latest Bangladesh demographic and health survey	Quantitative (survey)	Random and Women	MHC-ANC and childbirth	• Early marital age, early pregnancy, high parity, having on income, residing in rural area were identified as barriers for women seeking care	• Low parity, occupational involvement, high literacy, high wealth status and exposure to mass media were identified as enablers for women seeking ANC services
Saha et al. [37]	Rural-urban	To examine the socioeconomic factors associated with recommended, intermediate, and no ANC visits in Bangladesh using a conceptual behavioral model for healthcare services utilization for developing countries	Quantitative (survey)	Stratified, multistage cluster sample and Women	MHC-ANC	• High parity, low literacy, residing in rural area, and low income were barriers for women seeking care	• Low parity, high literacy, having a job, high wealth status, and exposure to mass media were facilitators for women to access the WHO recommended ANC visits
Rahman et al. [38]	Rural-urban	To investigate the association between maternal pregnancy intention and professional antenatal and delivery care utilization	Quantitative (survey)	Two-stage stratified cluster and Women	MHC-ANC	• Unwanted pregnancy, low literacy, high parity, having no income, low decision-making ability were identified as barriers for women seeking professional care	• High literacy, low parity, having income, and high wealth status were identified as enablers for women seeking care
Kamal et al. [39]	Rural-urban	To investigate the factors affecting the timing of ANC seeking among bangladeshi women using the 2007 Bangladesh demographic and health survey data	Quantitative (survey)	Multistage cluster sampling and Women	MHC-ANC	• Low literacy level, poorest wealth quintile, residing in rural area, and high birth order were identified as barriers to seeking ANC services by women	• High literacy level, richest wealth quintile, residing in urban area, and low birth order were identified as enablers for women seeking ANC services
Rai [40]	Rural-urban	To analyze the factors associated with utilization of maternal healthcare services among muslim women residing in each country	Quantitative (survey)	Multistage cluster sampling and Women	MHC-ANC	• Early pregnancy, high parity, low literacy, and residing in rural area were barriers for women in seeking care	• High literacy, low parity, and highest wealth quantile were associated with the utilization of MHC services by women
Sarker et al. [41]	Rural-urban	Examine the associations of socioeconomic determinants of women aged 12–49 years with the CCs awareness and visitation	Quantitative (survey)	Cluster sampling design and Married women	PHC and MHC	• Low education and young age were negatively associated with utilization of community clinics	• High education, adult age, and living in rural area were identified as facilitators
Rahman [42]	Rural-urban	To identify influential factors that are affecting maternal healthcare services and treatment seeking behavior	Quantitative (survey)	Not reported and Married women	MHC-ANC, PNC and childbirth	• Low literacy, poor wealth status, and no mass media exposure prevented women from seeking care	• High literacy, high wealth status, mass media exposure and NGO affiliation increased women’s MHC utilization
Chakraborty et al. [43]	Rural	Examine the factors that influence the maternal healthcare services in Bangladesh	Quantitative (survey)	Multistage random sampling and Pregnant women	MHC	• Early age of marriage and early age of being a mother prevented women from seeking timely care	• Women’s low level of education, and husband’s occupation have strong influence on healthcare utilization
						• Delays in family decision-making was also a barrier for seeking timely care	
Akter et al. [44]	Rural-urban	To assess ANC quality and identify the sociodemographic factors associated with the usage of quality ANC services in Bangladesh	Quantitative (survey)	Multistage stratified cluster sampling design and Pregnant women	ANC	• Residing in rural areas, having no education and media exposure, and having a high birth order reduced women to receive high-quality ANC services	• NP
Islam et al. [45]	Rural-urban	To examine the association between pregnancy intention and ANC-seeking behaviors among women in Bangladesh.	Quantitative (survey)	Two-stage stratified sampling and Women	ANC	• Early age of being a mother was related to unintended pregnancy	• NP
						• Unintended pregnancy was related to low utilization of ANC	
						• ANC utilization was lower among women with rural residence, low literacy level, and poorest wealth quantile	
Nizum et al. [46]	Rural	To assess the utilization and underlying factors affecting the utilization of ANC among women of reproductive age in rural Bangladesh.	Quantitative (survey)	Non-random and Women	ANC	• Low literacy, low age, low family income were barriers for women to access ANC services	• Women with more age, higher education, having more than five members in a family, and being involved in any income-generating activities were more likely to utilize ANC services
Hajizadeh et al. [47]	Rural-urban	To provide a comprehensive analysis of trends in social inequalities in utilization of ANC, FBD, and SBA in Bangladesh between 1995 and 2010	Quantitative (survey)	Not clearly reported and Women	MHC-ANC, FBD and SBA	• Living in rural areas, low level of education, and poverty were key barriers in seeking care	• Being wealthier, having higher education and, living in urban areas were facilitators for women in using MHC services
Paul and rumsey [48]	Rural	To identify determinants of the use of medical center and TBAs for delivery purposes in a rural area of Bangladesh	Quantitative (survey)	Not clearly reported and Women and men	MHC-childbirth	• Illiteracy and lack of prior ANC checkup prevented women from utilizing childbirth services	• High literacy and prior ANC checkup were identified as facilitators for women seeking care
Edmonds et al. [49]	Rural-urban	To test the predictive value of women’s self-identified criteria in place of birth decisions in the event of uncomplicated childbirth in a setting where facility based skilled birth attendants are available	Mixed methods (interview and survey)	Multistage sampling and Married women [18–49]	MHC-FBS	• Early age of marriage, and inadequate transportation affected women’s decision-making ability for childbirth	• Exposure to mass media was associated with health services utilization of women
Islam and odland [50]	Rural	To examine factors associated with antenatal and postnatal care visits among the *Mru*, the most underprivileged indigenous people in Bangladesh	Mixed methods (survey and interview)	Purposive and Married women	Maternal-ANC and PNC	• Travelling distance and poor transportation reduced women’s ability to timely access to ANC and PNC services	• Higher levels of literacy, job involvement and exposure to mass media helped women to seek ANC services
Khatun et al. [51]	Rural	Not clear	Qualitative (interview)	Purposive and General public, students, community leaders, school teachers, and formal and informal healthcare providers	mHealth for both PHC and MHC	• Inequality existed in using mHealth services based upon educational status	• mHealth services, were used to consult with a qualified doctor
							• mHealth also saved time and cost
Khanam et al. [52]	Rural	To identify the prevalence of antepartum and intrapartum complications and determinants of care seeking for these complications in rural Bangladesh	Quantitative (survey)	Not reported and Married women	MHC-antepartum and intrapartum complications	• Low literacy, low decision-making ability, long distance to facilities were identified as barriers to women seeking MHC services	• Belonging to wealthy families, having higher education, and living close to health facilities (<10 km) were facilitators for women seeking care for MHC services
Begum and hamid [53]	Rural	To explore the variation in ANC visits and institutional delivery between high and low disaster-prone areas	Quantitative (survey)	Multi-stage stratified sampling and Women	ANC and childbirth	• Identification of a very low utilization of 4+ ANC visits in both high (20%) and low (15%) disaster-prone areas	• Having higher education, large household size, high income, and proximity to health facilities were associated with women’s higher ANC visits
						• Identification of a difference in C-section use between high (42%) and low (79%) disaster-prone areas	
Khatun et al. [54]	Rural	To examine gender differences in awareness of mobile phone use for healthcare services and knowledge of available mHealth services	Quantitative (survey)	Random and Women and men	mHealth for MHC	• Women were less likely to own mobile phones than men to access mHealth services	• Women received mHealth services through shared mobile phones
						• Lack of women’s knowledge about using mHealth services	
Begum et al. [55]	Rural	To explore the attitudes of both women and obstetricians towards caesarean section birth in a rural area of Bangladesh	Qualitative (FG and interview)	Purposive and Women and obstetricians	MHC-ANC	• Women had the erroneous view that episiotomy itself is a ‘small caesarean’	• NP
						• Primary healthcare providers and clinic agents (brokers) had a strong influence on the decision of women in choosing a health facility for childbirth	
Hossain et al. [56]	Rural-urban	To investigate women’s pregnancy decision making process, reasons for their denial of MR, the barriers they confront in obtaining MR, and where they go after denial of MR.	Qualitative (interview)	Purposive and Women	MR services	• Identification of a lack of knowledge about the legal period limit for government-approved MR services of women	• Cleanliness and privacy of the clinic satisfied women
						• Service costs	
Alam et al. [57]	Rural	To understand community preparedness for IFA supplementation early in pregnancy and to inform the design of a large-scale trial of early introduction of IFA supplementation in rural Bangladesh	Qualitative (interview, KII, FG and observation)	Stratified purposive and opportunistic sampling and Pregnant women, older women, fathers, doctors, TBA, female college students	ANC	• A culturally informed perceived barrier was the belief that IFA supplementation will increase fetus size, leading to birth complications, hospitalization, C-section, and financial burden for families	• Taking IFA tablets during pregnancy makes women feel better
							• Women believed that the IFA increased blood volume, resulting in foetal nutrition and compensation for blood loss due to childbirth
Choudhury and ahmed [58]	Rural	To inform the program interventions which would hopefully improve maternal morbidity and survival and achieve the UN millennium development goal 5	Qualitative (interview)	Purposive and Women	MHC-ANC, childbirth and postpartum care	• Financial constraints, traditional beliefs and rituals delayed care-seeking of women	• Identification of support from husbands in seeking MHC care
Afsana and rashid [59]	Rural	To determine how to improve existing BHC services and create a new model of service delivery	Qualitative (interview, FG and informal discussion)	Purposive and Women aged 20–40 and TBAs	MHC-childbirth services	• Cost, fear, and the stigma of an ‘abnormal' childbirth	• Caring for childbirth
						• Most women during complications only attended the facility, where services were limited	• More capable of managing normal childbirth
Gazi et al. [60]	Rural-urban	To assess changes in knowledge among married women of reproductive age on selected reproductive health issues and to explore their service utilization patterns over the project period in selected low performing areas of Bangladesh	Quantitative (survey)	Simple random sampling and Women	MHC and reproductive health-ANC, PNC and family planning	• Misconception about pregnancy complications and fear in treatment prevented women seeking care	• Received free contraceptive methods from the public sectors
							• Received free ANC and PNC services
Sikder et al. [61]	Rural	To describe the healthcare decision-making process during severe acute obstetric complications among women and their families in rural Bangladesh	Qualitative (interview)	Purposive and Women	MHC-ANC	• Delay in decision-making by male	• Own decision-making ability for induced abortions
						• Inadequate transport	
						• High service costs	
						• Non-certified healthcare providers	
Shahjahan and Kabir [62]	Rural-urban	To assess males’ perception, attitude, and knowledge on reproductive health matters	Qualitative (FG)	Not clearly reported and Men	Reproductive	• Some sociocultural factors such as poor interaction between husbands and wives impeded spousal communication of reproductive health, which discouraged male to take their wives to health clinics	• NP
Rahman et al. [63]	Rural-urban	To explore the association between maternal experiences of physical and sexual IPV and the use of reproductive healthcare services, using a large nationally representative data set from Bangladesh	Quantitative (survey)	Stratified, multistage cluster sample and Married women	MHC-ANC	• About two of the four participants experienced physical IPV.	• NP
						• The mother’s experience with IPV was associated with a low use of ANC	
Aziz et al. [64]	Rural-urban	To analyze the trends, inequalities, and factors associated with PNC for mothers in Bangladesh	Quantitative (survey)	Stratified cluster-sampling and Women	PNC	• Giving birth at home, belonging to the lowest wealth quantile and receiving no ANC adversely affected women to receive timely PNC services	• Belonging to the highest wealth quintile increased the PNC rate of women
Amin et al. [65]	Rural	To examine the socioeconomic differentials in health-seeking behavior	Quantitative (survey)	Purposive and Women	MHC-PNC	• Belonging to the poorest wealth quintile reduced women’s ability to seek MHC services	• Belonging to the highest wealth quintile supported women to use ANC, childbirth, PNC, and child healthcare services from modern trained providers
						• Belonging to the poorest wealth quintile reduced women’s ability to make timely decisions	
Banik [66]	Rural-urban	Finding out physical, social and organizational access barriers to MHC services and exploring how these barriers caused three delays in healthcare seeking behavior	Mixed methods (survey, interview and FG)	Multistage sampling and Women	MHC	• Social barriers to MHC services were early marriage, perception of pregnancy and childbirth, and cost	• NP
						• Organizational barriers included lack of female healthcare staff, lack of guidelines and low-quality services	
						• Physical barrier included distance	
Orderud et al. [67]	Rural-urban	To analyze associations between flood exposure and the use of maternal healthcare in Bangladesh	Quantitative (survey)	Two-stage cluster sampling and Women	MHC	• Pregnant women living in the area exposed to flooding faced challenges in using MHC services	• NP
Adhikary et al. [68]	Rural-urban	To measure the level of patients’ satisfaction across different types and levels of healthcare facilities and to determine which factors influence this satisfaction level	Quantitative (survey)	Purposive and Women and men	MHC	• Uncleanliness, and lack of privacy settings in public facilities	• Facilitators in private facilities included convenient opening hours, privacy, and cleanliness
						• Long distance to reach private facilities	
Akhter et al. [69]	Rural-urban	To present findings from the qualitative study highlighting barriers and facilitators for obtaining emergent blood from the perspectives of HPs, attendants and unlicensed blood brokers	Qualitative (interview and KII)	Purposive and snowball sampling and Hospital directors, managers, providers, blood bank authorities, unlicensed blood brokers, and patients’ relatives	MHC- safe blood transfusion	• Heavily dependent on a network of unlicensed blood brokers during emergencies	• The new online system facilitated blood transfusion processes at any time for poor patients at a low cost
Rob and Alam [70]	Rural-urban	To examine the impact of performance-based incentives for service providers at the institutional level to improve the quality of maternal health services	Quantitative (survey)	Not clearly reported and Healthcare providers	MHC-ANC and PNC	• NP	• Incentives to healthcare providers increased the quality of care
							• Free services and medicine increased patients’ satisfaction

MHC: maternal healthcare, FG: focus group, BHC: BRAC healthcare, HP: healthcare provider, KII: key informant interview, TBA: traditional birth attendant, ANC: antenatal care, NP: not provided, IFA: iron and folic acid, PNC: postnatal care, FBS: Facility-Based Service, FBD: Facility-Based Delivery, SBA: skilled birth attendant, MR: menstrual regulation, Km: Kilometer, CS: cesarean section, IPV: intimate partner violence, WHO: world health organization, CC: community clinic.

## Results

### Study Characteristics

Table 2 presents a summary of the characteristics of the studies included in this review. Studies were published between 2001 and 2023. The majority of studies were published between 2011 and 2018 (n = 23; 62%). Most of the included studies (n = 29; 78%) in this review were considered of high quality, and the remaining eight were of medium quality (Table 2). The review included 37 studies, of which 24 were quantitative, 10 were qualitative, and three were MM studies. Fourteen studies were carried out in rural locations, and the remaining 23 studies focused on both rural and urban locations.

### Description of Themes

Four key themes related to accessing and using MHC services by rural Bangladeshi women emerged from the thematic analysis of the studies, using the SEM as the guiding framework: (i) individual-level barriers and facilitators; (ii) family-level barriers and facilitators; (iii) community and social-level barriers and facilitators; and (iv) organizational-level barriers and facilitators.

### Theme 1: Individual-Level Barriers and Facilitators to Accessing and Using MHC Services

This theme included two sub-themes, including demographic barriers and facilitators and lack of self-confidence in treatment.

### Demographic Barriers and Facilitators

Fifteen quantitative [34–48], two MM studies [49, 50], and one qualitative [51] out of the 37 studies focused on demographic barriers to women accessing MHC services. Women and their husbands with lower levels of education [34, 35, 37–41, 43–49, 51, 52] reported having less access to MHC services. Educated mothers were approximately 16 times more likely to receive ANC compared to illiterate mothers, and 14 times more likely to obtain the components of ANC content visits, such as counselling regarding danger signs [35].

Another barrier is related to the early age of being married and being a mother of women. Women living in rural Bangladesh reported that early age of marriage [35, 36, 38, 43, 49] and early age of being a mother [36, 38–41, 43, 45, 46] impeded them from accessing and using MHC services. Women aged 30–49 in rural Bangladesh reported that they had twice as many facility-based deliveries compared to the younger group [36]. In addition, higher parity (the number of children a woman has had) [34, 36–41, 44, 46], mothers’ involvement with no income-generating activities [34, 36, 38, 43, 46], unintended pregnancy [38, 45], and lack of ANC checkup [48] were also identified as barriers to accessing and using MHC services for women living in rural Bangladesh.

Positive associations between demographic facilitators and access to and use of MHC services were identified in 16 quantitative studies [34, 36–41, 43–48, 52–54] and one MM [50]. Four facilitators were found to be associated with access to and use of MHC services by women: higher literacy level enabled women to be aware of the MHC services [38, 40, 41, 43–48, 52–54]; low parity [35–37, 40, 44]; having income made women capable of affording the cost associated with services [46, 50, 53]; and the mother being older showed greater experience of seeking services [36, 38]. For example, ANC visits were found to be three times higher among mothers with a higher level of education compared to mothers with no education [35].

### Lack of Women’s Self-Confidence in MHC Treatment

Ten studies, five qualitative [55–59] and five quantitative [34, 38, 52, 54, 60], reported on the lack of self-confidence in MHC treatment among women living in rural Bangladesh. This lack of self-confidence was based on a number of factors. Misconceptions or limited knowledge of MHC services [54–57, 59, 60] were reported as the reason for women’s self-confidence in treatment. In most cases, women living in rural areas of Bangladesh were not informed about the reasons for their physical examinations [55, 59], taking medicines [57], and menstrual regulation (MR) services [56, 59], which led to misconceptions among them. Additionally, fear in using MHC services [55–60] was identified as being linked with women’s lack of self-confidence. Fear was mostly reported to be associated with pain, increased blood pressure after childbirth, etc. [55, 59, 60]. Finally, low decision-making ability [34, 38, 52] was identified as a barrier to women residing in rural Bangladesh in seeking treatment.

### Theme 2: Family-Level Barriers and Facilitators to Accessing and Using MHC Services

Thirteen studies, six qualitative [55, 57–59, 61, 62], five quantitative [43, 47, 63–65], and two MM [49, 66], reported on family issues that were reported to hinder women in accessing and using MHC services. As reported in several studies, delays in decision-making when seeking MHC services were identified as a family-level barrier, as women from rural Bangladeshi families relied on family members, including husbands and mothers-in-law, for seeking advice to travel outside that is often delayed [43, 49, 55, 57, 59, 61, 65]. Moreover, absence of husbands’ participation in maternity care due to their shyness and family culture [62], intimate partner violence (IPV) [63], family financial constraints to afford the expense related to using MHC services [47, 58, 64], and a culture of home childbirth [64, 66] were commonly reported as family-level barriers to women accessing and using MHC services.

However, 15 studies, 13 quantitative [34–40, 43, 44, 52, 54, 64, 65], one qualitative [58] and one MM [50], highlighted how the family supported women’s access to MHC services. Husbands in a patriarchal society like Bangladesh generally have a key household decision-making role. Husbands who had a higher level of education [34, 35, 37, 40, 52], occupational involvement [36, 37, 43, 50], wealth status [35–39, 44, 52, 64, 65], positive attitudes toward care [58, 59], and shared their mobile phones with women to access mHealth services [54] were found to be positively related to women’s access to and use of MHC services.

### Theme 3: Community and Social-Level Barriers and Facilitators to Accessing and Using MHC Services

Three subthemes were identified under this theme: sociocultural barriers, geographical barriers, and Non-Governmental Organization (NGO) and microcredit support and mass media exposure.

### Sociocultural Barriers

Several sociocultural barriers were highlighted in six studies, which include four qualitative [55, 57, 58, 62], one quantitative [54], and one MM [66]. Traditional attitudes and beliefs about pregnancy and childbirth [55, 57, 58, 62, 66] were identified as a sociocultural barrier for women living in rural Bangladesh to access and use MHC services. For example, there was a belief that pregnancy disclosure could lead to unwanted spiritual complications and that pregnant women going outdoors during certain times of the day and week was considered to be harmful to the fetus [57]. Additionally, gender inequality in mobile phone ownership affected women, as they were unable to make a call to a doctor if needed [54]. Finally, religious rituals such as setting up a separate room outside of the household for childbirth and seclusion [58, 66] were reported to adversely affect women residing in rural Bangladesh in accessing and using facility-based delivery care.

### Geographical Barriers

Geographical barriers were reported in 14 studies, which included 10 quantitative [35, 36, 39, 40, 44, 45, 47, 52, 53, 67], three MM [49, 50, 66], and one qualitative [61]. Geographical barriers included residing in rural areas, which reduced women’s access to MHC due to limited and remote healthcare facilities [35, 36, 39, 40, 44, 45, 47]. For example, urban women, as a result of having available healthcare facilities, were 1.4 times more likely to receive ANC services compared with their rural sisters (OR = 1.351; 95% CI: 1.104–1.496) [35]. Another geographical barrier included long travel distances to healthcare points, which prevented women from seeking care, as it involved physical risks to pregnant women [50, 52, 66]. Moreover, having inadequate transportation [49, 50, 61] and living in a flood-prone area [53, 67] were all found to be associated with a delay in seeking MHC services among women living in rural Bangladesh.

### NGO and Microcredit Support and Mass Media Exposure

Seven studies, five quantitative [35–37, 42, 44] and two MM [49, 50], reported on NGO and microcredit support and/or mass media exposure. Three studies [35, 42, 50] reported that NGO and microcredit involvement among Bangladeshi women empowered them to access and use MHC services. For example, utilization of MHC services was found to be twice as high among mothers who visited NGO health facilities as compared to those who did not use the facilities [35]. Additionally, six studies reported that exposure to mass media (television and radio) messages [35–37, 42, 44, 49] was helpful for women in accessing healthcare services. For example, awareness-raising messages, such as timely using ANC services at the nearest community clinic and risks of early conception, through television or radio programs have been found to be effective by expectant mothers who reside in rural Bangladesh [36, 37].

### Theme 4: Organizational-Level Barriers and Facilitators to Accessing and Using MHC Services

Eight studies, five qualitative [55, 56, 58, 59, 61], two quantitative [48, 68] and one MM [66], focused on organizational barriers to access and utilization of MHC services by women living in rural Bangladesh. A wide range of organizational-related barriers were identified. An important organizational barrier included lack of quality healthcare services [55, 66], which affected women using satisfactory services. Patients in rural Bangladesh reportedly received poorer quality of MHC services for the following reasons: shortages of qualified personnel, lack of training of health staff, and limitations of diagnosis [55, 66]. Experiencing mistreatment, including use of vulgar words, from healthcare providers [48, 55, 58, 59] was also reported to be another important organizational barrier to assessing and using MHC services for women living in rural Bangladesh. Moreover, the gender of the healthcare personnel [59, 66] was reported to be an issue when women felt discomfort seeking care from male doctors. Another barrier included service costs related to medication and investigation fees [56, 58, 61, 66], discriminatory service provision [66], and uncleanliness and lack of privacy [68].

Organizational facilitators were the factors that helped women to access and use of MHC services in public and private facilities, identified in four qualitative [51, 57, 59, 69] and three quantitative [54, 60, 70] studies. Key facilitators to increasing access and use of MHC services by women in public facilities included: (i) health center-based support, including free services to users [57, 60, 69, 70]; (ii) incentives to healthcare providers [70]; and (iii) technology support, including mHealth such as calling doctors from the home or online services such as emergency blood management [51, 54, 69] to reduce the cultural gap between the patient and doctor to the use of services. Additionally, one quantitative [68] and two qualitative [56, 59] studies reported on some private health center-based facilitators, that included convenient opening hours, privacy, cleanliness, caregiving, and normal childbirth management skills. Women were reported to be more satisfied with private facilities compared to public facilities.

## Discussion

This narrative literature review aimed to identify and critically synthesize published studies examining barriers and facilitators to women living in rural Bangladesh in accessing and using MHC services. This review has identified a number of barriers and facilitators influencing the access to and the utilization of MHC services by women residing in rural Bangladesh. These findings have been categorized using the SEM as individual, family, community and social, and organizational levels of health influence, as it is recognized that human behavior is influenced and shaped by multiple, interrelated factors [17, 18].

This narrative review has identified that at the individual-level, education and literacy levels, among both women and their husbands, were found to be the most identified factor related to access and utilization of ANC services among women residing in rural areas in Bangladesh. For instance, women and their partners are more likely to access and use of MHC services when their education and literacy levels are satisfactory, as compared to individuals with lower levels of these factors. This supports previous research in the area that suggested an increase in knowledge and health literacy among women residing in rural areas [71–73]. Although improving knowledge remains an effective intervention in rural settings to increase access and utilization of MHC services by women residing in rural settings, it is also important to consider the husbands’ knowledge levels in this area [72].

The findings also suggested that women’s lack of self-confidence in their treatment, due to a lack of health literacy, fear of treatment, etc., was a further barrier to accessing and using MHC services. This finding presents a new issue, as it was not highlighted in the previous review [14] in the MHC field in Bangladesh. This finding corroborates and builds on previous research [74], where it was identified that women did not access the facility-based childbirth as a result of a lack of awareness. Therefore, educational interventions need to include increasing knowledge and health literacy skills regarding the need for access and use of MHC services through raising awareness programs for both women and their husbands.

At the family-level, decision-making to seek MHC care was found to be an influencing factor in both access and use of MHC services is crucial, as women’s healthcare was dependent on timely decision-making. However, this review identified that for women living in rural Bangladesh, healthcare decision-making was mainly found to be based on family members’ views, which can lead to delays in seeking care. This finding builds on the findings of studies published in Bangladesh [75, 76] and Pakistan [77], where senior and/or male family members were mostly reported to decide family issues, including healthcare. In a patriarchal society like Bangladesh, husbands play a dominant decision-making role in all family aspects, including healthcare expenditure, purchasing household items, etc., as they are mostly the income earners [76]. Additionally, the findings from this review identified that some husbands, who lived in rural areas in Bangladesh, had less participation in women’s maternity care, which has added a new finding, and it was not reflected in the previous review [14]. Two factors were identified in the review literature that were related to men’s low participation in women’s MHC services: (i) negative perceptions of a patriarchal society and (ii) restrictions on men’s access to maternity clinics [78–80]. WHO [81] recommends that the participation of men as an intervention during maternity care can enable women to make decisions about their healthcare and that of their newborns. Also, women who were physically and sexually abused by their partner were reported to be less likely to have visited a qualified ANC provider. This is possibly as a result of women’s stress after experiencing abuse, which could lead to a lack of motivation in pursuing appropriate care [63]. Therefore, increasing women’s decision-making power and involving their partners in MHC services could increase accessing and using MHC services by women in rural Bangladesh, which are also suggested in other studies [75–77].

The narrative review identified some negative social and cultural practices, such as traditional attitudes and beliefs about pregnancy and childbirth, which were revealed as barriers to women both accessing and using MHC services at the community and societal-level. This supports previous research in the area conducted in South Asia [82, 83] that suggested pregnant South Asian women who resided in rural areas preferred home delivery over facility-based delivery due to privacy concerns and an unwillingness to divulge their pregnancy [84, 85]. However, these concerns could contribute to an increase in high maternal mortality and other maternal complications [85]. Therefore, developing culturally sensitive interventions with women around increasing awareness of the importance of facility-based delivery are needed to increase access and use of MHC services by women living in rural Bangladesh. Another, the distance needed to travel to health center facilities by women living in rural Bangladesh, is often reported a as barrier identified in the review and seen in previous research studies [77, 82, 84]. Constructing village roads and strengthening mHealth services can be the solution to reduce the distance barrier.

Rural dwelling Bangladeshi women’s connection with NGOs and microcredit connections was found to increase their empowerment levels around informed decision-making, being more likely to access health information and healthcare services than those without such connections. This may be due, in part, to the fact that NGOs have their own health centers for their clients, and that the services are less costly and more easily accessible. This supports previous research in the area conducted in African and South Asian countries for women seeking access and use of ANC services [86, 87]. This narrative literature review adds this new information, which is different from the previous review undertaken on MHC services in 2011 [14]. These studies also identified that, when women received loans from NGOs and microcredit organizations, they utilized that money to get out of poverty, which ultimately increased their capability of accessing MHC services. Additionally, women’s wider social networks beyond their family were identified as being supportive and a useful resource for women with regard to receiving health-related information from organizations or neighbors. Thus, the connection with NGOs and microcredit organizations was found to be helpful for women living in rural Bangladesh to become more independent in accessing and using MHC services.

The narrative review found that, at the organizational-level, there was a reported lack in the quality-of-service provision, which was identified as a major barrier for both access and use of MHC services. Although most women in the reviewed studies were found to frequently visit government-run health centers, these studies also highlighted that women were more satisfied with privately-run services. Women’s dissatisfaction with government facilities was found to be due to its poor service provision such as a lack of quality healthcare services, which supports previous studies in the area [88, 89]. The review also identified that women also experienced and/or reported mistreatment, including rude behavior, late surgery appointments, and physical abuse by doctors and/or nurses whilst using MHC services. Such mistreatment does not coincide with the National Health Policy of Bangladesh [90] and the United Kingdom National Institute for Health and Care Excellence (NICE) guidelines [91], which emphasize that advice and information should be provided to women about specific treatments and care options. Although, based on this review, women’s experience of existing MHC services was found to be mixed, undesired experiences were more evident at each stage of receiving services. Therefore, MHC staff need further education and training to support them in improving women’s access and use of MHC services by the provision of satisfactory services. This recommendation is supported by previous research in the area that was conducted in Asia and Africa, which found that health workers needed adequate MHC service provision-related education and training to provide satisfactory services to women living in rural Bangladesh [92, 93].

A supportive organizational culture such as free service, mostly in public health centers, was identified as a facilitator for women accessing and using MHC services, and is supported by previous research in the area [77]. However, clinical investigations are not free and sometimes unavailable, making them costly outside. Therefore, to improve MHC services use, the number of items, including medicines, beds, and investigation items, should be covered under free or reduced-price services, especially for the women residing in rural Bangladesh.

### Strengths and Limitations

Although one review on access to healthcare services by women in rural Bangladesh [14] has been published, it was conducted more than a decade ago. This is the first known narrative literature review with a systematic search and updated study to identify access to and use of MHC services by women in rural Bangladesh. This review’s strength lies in its application of the SEM model to synthesize findings spanning more than 20 years of literature on the barriers and facilitators to accessing and utilizing MHC services by women in rural Bangladesh.

However, there are some limitations to this narrative literature review. There are not many studies on health-related topics published in Bengali, and most of them lack peer review, so their potential impact is likely to be minimal. Therefore, the review only included peer-reviewed studies and those published in English due to limited resources, which may have narrowed the scope of this review. It is likely that studies in other languages, particularly Bengali, may have provided further contributions. In addition, the wide variety of study methods precluded the possibility of conducting a meta-analysis, which can be used to identify the common effect [5].

### Conclusions

Accessing and using MHC services for women residing in rural Bangladesh is a complex issue involving a wide range of barriers and facilitators at the individual, family, community and social, and organizational levels. By using the SEM framework to guide this review, the influence of multiple, interrelated factors in shaping behavior to access and use MHC services by women living in rural Bangladesh has been highlighted. More studies focusing on the barriers than the facilitators were identified in this review. Significant barriers to accessing and using MHC services encompass low levels of women’s literacy, insufficient self-confidence regarding treatment, delays in decision-making processes, absence of male support, a preference for home childbirth, restrictions on disclosing pregnancy, distance to healthcare facilities, and inadequate quality of care. Conversely, key facilitators, such as high literacy rates of women and their spouses, support from NGOs and microcredit programs, media exposure, and the availability of free services, enhance women’s access to and use of MHC services in rural areas. To attain SDG3, policymakers and healthcare professionals need to focus on these barriers and facilitators and develop interventions that can increase access and use of MHC services by women residing in rural Bangladesh. Researchers can use these findings to help make plans for how to enable more women in low- and middle-income countries like Bangladesh to access and use MHC services. Further studies, including meta-analyses are needed to understand the causal relationship and actual magnitude of the barriers and facilitators to MHC services.
