# Supplementary material for: Barriers and Facilitators to Accessing and Using Maternal Healthcare Services by Women Living in Rural Bangladesh: A Theory-Guided Narrative Literature Review
Source: Public Health Rev. 2025 Dec 29;46:1608157. doi: 10.3389/phrs.2025.1608157 (PMC12791047; doi:10.3389/phrs.2025.1608157)
Supplement: Supplementary file 1 [file Table1.docx]

**TABLE |** Search strategies with the MEDLINE (Ovid) database.

| **#** | **Search terms** | **Results** |
| --- | --- | --- |
| 1 | (barrier* or discriminat* or difficult* or challenge*).mp. [mp=title, abstract, original title, name of substance word, subject heading word, floating sub-heading word, keyword heading word, organism supplementary concept word, protocol supplementary concept word, rare disease supplementary concept word, unique identifier, synonyms] | 1321585 |
| 2 | (facilitator* or motivat* or enabler*).mp. [mp=title, abstract, original title, name of substance word, subject heading word, floating sub-heading word, keyword heading word, organism supplementary concept word, protocol supplementary concept word, rare disease supplementary concept word, unique identifier, synonyms] | 151817 |
| 3 | 1 and 2 | 28868 |
| 4 | "Delivery of Health Care"/ | 83390 |
| 5 | Health Services Accessibility/ | 68216 |
| 6 | "Patient Acceptance of Health Care"/ | 41672 |
| 7 | (health care access* or healthcare access* or community health care* or antenatal care* or postnatal care* or primary health care* or medical care*).mp. [mp=title, abstract, original title, name of substance word, subject heading word, floating sub-heading word, keyword heading word, organism supplementary concept word, protocol supplementary concept word, rare disease supplementary concept word, unique identifier, synonyms] | 137815 |
| 8 | (healthcare* utili#ation or health care utili#ation or health care application* or health care employment* or health care practice* or health care operation*).mp. [mp=title, abstract, original title, name of substance word, subject heading word, floating sub-heading word, keyword heading word, organism supplementary concept word, protocol supplementary concept word, rare disease supplementary concept word, unique identifier, synonyms] | 10485 |
| 9 | 4 or 5 or 6 or 7 or 8 | 306831 |
| 10 | Women/ | 14437 |
| 11 | (women or female).mp. [mp=title, abstract, original title, name of substance word, subject heading word, floating sub-heading word, keyword heading word, organism supplementary concept word, protocol supplementary concept word, rare disease supplementary concept word, unique identifier, synonyms] | 8283616 |
| 12 | 10 or 11 | 8283616 |
| 13 | Bangladesh/ | 9586 |
| 14 | (Bangladesh* or India or Pakistan or Nepal or Bhutan or South Asia or developing countr* or low-and middle-income countr*).mp. [mp=title, abstract, original title, name of substance word, subject heading word, floating sub-heading word, keyword heading word, organism supplementary concept word, protocol supplementary concept word, rare disease supplementary concept word, unique identifier, synonyms] | 247513 |
| 15 | 13 or 14 | 247513 |
| 16 | 3 and 9 and 12 and 15 | 187 |
| 17 | limit 16 to (english language and female and full text and humans and english and medline) | 41 |
| 18 | limit 17 to "all adult (19 plus years)" | 23 |
