# Supplementary material for: Barriers and Facilitators to Accessing and Using Maternal Healthcare Services by Women Living in Rural Bangladesh: A Theory-Guided Narrative Literature Review
Source: Public Health Rev. 2025 Dec 29;46:1608157. doi: 10.3389/phrs.2025.1608157 (PMC12791047; doi:10.3389/phrs.2025.1608157)
Supplement: Supplementary file 2 [file Table2.docx]

**TABLE |** Appraisal tools for studies included in this review.

| Quantitative cohort studies (Y= *yes*, N= *no*, C= *can’t tell)* | | | | | | | | | | | | | | | |
| --- | --- | --- | --- | --- | --- | --- | --- | --- | --- | --- | --- | --- | --- | --- | --- |
| Author, year, country | 1. Did the study address a  clearly focused issue? | 2. Was the cohort recruited in an acceptable way? | 3. Was the exposure accurately measured to minimise bias? | 4. Was the outcome accurately measured to minimise bias? | 5. (a) Have the authors identified all important confounding factors? | 5. (b) Have they taken account of the confounding factors in the design and/or analysis? | 6. (a) Was the follow up of subjects complete enough? | 6. (b) Was the follow up of subjects long enough? | 7. What are the results of this study? | 8. How precise are the results? | 9. Do you believe the results? | 10. Can the results be applied to local population? | 11. Do the results of this study fit  with other available  evidence? | 12. What are the implications of  this study for practice? | Total score (%) and quality |
| 1. Chakraborty   et al., 2003,  Bangladesh | Y | Y | Y | Y | Y | Y | Y | Y | Exposure has impact on outcome | Very precise | Y | Y | Y | Y | 12 (100.0)  High |
| 1. Khanam et al., 2016   Bangladesh | y | y | y | y | y | C | y | y | Exposure has impact on outcome | Precise | y | y | y | y | 11 (92.0)  High |

| Quantitative cross-sectional studies (Y= *yes*, N= *no*, C= *can’t tell)* | | | | | | | | | | | | | |
| --- | --- | --- | --- | --- | --- | --- | --- | --- | --- | --- | --- | --- | --- |
| Author(s), year, country | 1. Did the study address a  clearly focused issue? | 2. Is the research method (study design) appropriate for answering the research question? | 3. Is the method of selection of the subjects (employees, teams, divisions, organizations) clearly described? | 4. Could the way the sample was obtained introduce (selection) bias? | 5. Was the sample of subjects representative with regard to the population to which the findings will be referred? | 6. Was the sample size based on pre-study considerations of statistical power? | 7. Was a satisfactory response rate achieved? | 8. Are the measurements (questionnaires) likely to be valid and reliable? | 9. Was the statistical significance assessed? | 10. Are confidence intervals given for the main results? | 11. Could there be confounding factors that haven’t been accounted for? | 12. Can the results be applied to your organisation? | Total score (%) and quality |
| 1. Adhikary et al., 2017, Bangladesh | Y | Y | Y | Y | Y | Y | Y | Y | Y | Y | Y | Y | 12 (100.0) High |
| 1. Akter et al., 2023, Bangladesh | Y | Y | Y | Y | Y | Y | Y | Y | Y | Y | Y | Y | 12 (100.0) High |
| 1. Amin et al.,   2010  Bangladesh | Y | Y | Y | C (No information about sampling bias is included.) | Y | C (This paper does not contain any information about pre-study considerations of statistical power.) | Y | Y | Y | Y | Y | Y | 10 (83.0)  High |
| 1. Aziz et al., 2022, Bangladesh | Y | Y | Y | Y | Y | Y | Y | Y | Y | Y | Y | Y | 12 (100.0) High |
| 1. Begum and Hamid, 2023, Bangladesh | Y | Y | Y | Y | Y | Y | Y | Y | Y | Y | Y | Y | 12 (100.0) High |
| 1. Hajizadesh et al. 2014, Bangladesh | Y | Y | Y | C (No information about sampling bias is included.) | Y | C (This paper does not contain any information about pre-study considerations of statistical power.) | Y | Y | Y | N (No CI has been used but P value is used with %.) | Y | Y | 9 (75.0)  Medium |
| 1. Huda et al.,   2018, Bangladesh, Nepal & Pakistan | Y | Y | Y | C | Y | C (Pre study considerations of statistical power related information is missing.) | Y | Y | Y | Y | C | Y | 9 (75.0)  Medium |
| 1. Islam et al., 2023, Bangladesh | Y | Y | Y | Y | Y | Y | Y | Y | Y | Y | Y | Y | 12 (100.0) High |
| 1. Islam and Masud,   2018  Bangladesh | Y | Y | Y | C | Y | C | Y | Y | Y | Y | Y | Y | 10 (83.0)  High |
| 1. Kamal et al. 2015, Bangladesh | Y | Y | Y | C (No information about biasness is described.) | Y | C (Pre study considerations of statistical power related information is missing.) | Y | Y | Y | Y | Y | Y | 10 (83.0)  High |
| 1. Khatun et al. 2017, Bangladesh | Y | Y | Y | C (No information about biasness is described.) | Y | Y | C (No information about biasness is described.) | Y | Y | Y | Y | Y | 10 (83.0)  High |
| 1. Nizum et al., 2023, Bangladesh | Y | Y | Y | Y | Y | Y | Y | Y | Y | Y | Y | Y | 12 (100.0) High |
| 1. Orderud et al., 2022, Bangladesh | Y | Y | Y | Y | Y | Y | Y | Y | Y | Y | Y | Y | 12 (100.0) High |
| 1. Paul and Rumsey, 2002, Bangladesh | Y | Y | Y | C (No information about biasness is described.) | Y | C (Pre study considerations of statistical power related information is missing.) | C (No information about biasness is described.) | Y | Y | N (Although CI was not included, odd ratio has been applied.) | Y | Y | 8 (67.0)  Medium |
| 1. Rahman et al. 2018, Bangladesh | Y | Y | Y | Y (Recall bias has been reported.) | Y | C (This paper does not contain any information about pre-study considerations of statistical power.) | Y | Y | Y | Y | Y | Y | 11 (92.0)  High |
| 1. Rahman et al. 2012,   Bangladesh | Y | Y | Y | C (No information about biasness is described.) | Y | C (Although this study was done based on national data, the sample size based on pre-study consideration was not outlined here.) | Y | Y | Y | Y | Y | Y | 10 (83.0)  High |
| 1. Rahman, 2009   Bangladesh | Y | Y | Y | C | C | C | Y | Y | Y | N | Y | Y | 8 (67.0)  Medium |
| 1. Rai, 2015, India, Bangladesh and Pakistan | Y | Y | Y | Y (Recall bias has been reported.) | Y | C (This paper does not contain any information about pre-study considerations of statistical power.) | Y | Y | Y | Y | Y | Y | 11 (92.0)  High |
| 1. Saha et al. 2015, Bangladesh | Y | Y | Y | C (No information about sampling bias is included.) | Y | C (No information about sampling bias is included.) | Y | Y | Y | Y | Y | Y | 10 (83.0)  High |
| 1. Sarker et al., 2015   Bangladesh | Y | Y | Y | C | Y | C | Y | Y | Y | Y | Y | Y | 10 (83.0)  High |

| Randomised Control Trail (RCT) studies (Y= *yes*, N= *no*, C= *can’t tell)* | | | | | | | | | | | | |
| --- | --- | --- | --- | --- | --- | --- | --- | --- | --- | --- | --- | --- |
| Author(s), year, country | 1. Did the study address a clearly focused issue? | 2. Was the assignment of  patients to treatments randomised? | 3. Were all of the patients  who entered the trial  properly accounted for at its conclusion? | 4. Were patients, health  workers and study personnel ‘blind’ to treatment? | 5. Were the groups similar at the start of the trial? | 6. Aside from the experimental  intervention, were the groups treated equally? | 7. How large was the treatment effect? | 8. How precise was the estimate of the treatment  effect? | 9. Can the results be applied to  the local population, or in your context? | 10. Were all clinically important  outcomes considered? | 11. Are the benefits worth the  harms and costs? | Total score (%) and quality |
| 1. Gazi et al. 2014, Bangladesh | Y | Y | Y | Y | Y | Y | A higher proportion of the women in endline compared to baseline used MHC services and birth control techniques. | The interventions improved several important RH indicators | Y | Y | N | 8 (89.0)  High |
| 1. Rob and Alam, 2014, Bangladesh | Y | Y | Y | C (This paper does not include such type of information.) | Y | C (This paper does not include such type of information.) | Services of ANC, childbirth, and PNC were measured. The facility using rate increased by 23%% in 14 months following the intervention facilities. | Performance-based incentive has the potential for utilising services. | Y | Y | C (This paper does not include such type of information.) | 6 (67.0)  Medium |

| Qualitative studies (Y= *yes*, N= *no*, C= *can’t tell)* | | | | | | | | | | | |
| --- | --- | --- | --- | --- | --- | --- | --- | --- | --- | --- | --- |
| Author(s), year, country | 1. Was there a clear statement of the aims of the research? | 2. Is qualitative  methodology  appropriate? | 3. Was the research  design appropriate to address the aims of the research? | 4. Was the recruitment  strategy appropriate to the aims of the  research? | 5. Was the data collected in a way that addressed the research issue? | 6. Has the relationship  between researcher and  participants been  adequately considered? | 7. Have ethical issues been  taken into consideration? | 8. Was the data analysis  sufficiently rigorous? | 9. Is there a clear statement  of findings? | 10. How valuable is the  research? | Total score (%) and quality |
| 1. Afsana and Rashid, 2001, Bangladesh | Y | Y | Y | Y | Y | Y | C (No ethical statement is reported) | Y | Y | High | 8 (89.0)  High |
| 1. Akhter et al., 2016, Bangladesh | Y | Y | N (In methodology, this paper shows of using MM, but only qualitative data were analysed and discussed.) | Y | Y | Y | Y | Y | Y | High | 8 (89.0)  High |
| 1. Alam et al, 2015, Bangladesh | Y | Y | Y | Y | Y | Y | Y | Y | Y | High | 9 (100.0)  High |
| 1. Begum et al. 2018, Bangladesh | Y | Y | Y | Y | Y | Y | Y | Y | Y | High | 9 (100.0)  High |
| 1. Choudhury and Ahmed, 2011, Bangladesh | Y | Y | Y | Y | Y | Y | Y | Y | Y | High | 9 (100.0)  High |
| 1. Hossain et al, 2016, Bangladesh | Y | Y | Y | Y | Y | Y | Y | Y | Y | High | 9 (100.0)  High |
| 1. Khatun et al. 2016, Bangladesh | N | Y | Y | Y | Y | C | N | Y | Y | High | 7 (78.0)  Medium |
| 1. Rahman et al., 2016, rural, Bangladesh | Y | Y | Y | Y | Y | Y | N | Y | Y | High | 8 (89.0)  High |
| 1. Shahjahan and Kabir, 2006, Bangladesh | Y | Y | Y | C | Y | C | N | Y | Y | Good | 6 (67.0)  Medium |
| 1. Sikder et al., 2011, Bangladesh | Y | Y | Y | Y | Y | Y | Y | Y | Y | High | 9 (100.0)  High |

| Mixed methods studies (Y= *yes*, N= *no*, C= *can’t tell)* | | | | | | | | | | | | | | | | | | |
| --- | --- | --- | --- | --- | --- | --- | --- | --- | --- | --- | --- | --- | --- | --- | --- | --- | --- | --- |
| Author(s), year & country | Screening questions | | 1. Qualitative studies | | | | | 2. Quantitative studies | | | | | 3. Mixed methods studies | | | | | Total score (%) and quality |
|  | S1. Are there clear research questions? | S2. Do the collected data allow to address the research questions? | 1.1. Is the qualitative approach appropriate to answer the research question? | 1.2. Are the qualitative data collection methods adequate to address the research question? | 1.3. Are the findings adequately derived from the data? | 1.4. Is the interpretation of results sufficiently substantiated by data? | 1.5. Is there coherence between qualitative data sources, collection, analysis and interpretation? | 2.1. Is the sampling strategy relevant to address the research question? | 2.2. Is the sample representative of the target population? | 2.3. Are the measurements appropriate? | 2.4. Is the risk of nonresponse bias low? | 2.5. Is the statistical analysis appropriate to answer the research question? | 3.1. Is there an adequate rationale for using a mixed methods design to address the research question? | 3.2. Are the different components of the study effectively integrated to answer the research question? | 3.3. Are the outputs of the integration of qualitative and quantitative components adequately interpreted? | 3.4. Are divergences and inconsistencies between quantitative and qualitative results adequately addressed? | 3.5. Do the different components of the study adhere to the quality criteria of each tradition of the methods involved? |  |
| 1. Banik, 2016 Bangladesh | Y | Y | Y | Y | Y | Y | Y | Y | N (The study did not clarify how 160 samples were representative. Also, it was not clear if any selected respondent declined to respond.) | Y | C | N (There was no explanation to judge if descriptive statistics are appropriate for the design and research question.) | Y | Y | Y | Y | N | 13 (76.0)  Medium |
| 1. Edmonds et al. 2012 Bangladesh | Y | Y | Y | Y | Y | Y | C (This paper did not focus qualitative data analysis as qualitative phase was used to set of discrete decision criteria and then used to create question.) | Y | Y | Y | Y | Y | Y | Y | Y | N (This paper did not focus divergences and inconsistencies as qualitative phase was used to set of discrete decision criteria and then used to create question.) | Y | 15 (88.0)  High |
| 1. Islam and Odland, 2011 Bangladesh | Y | Y | Y | Y | Y | Y | N (Data interpretation was not mainly based on qualitative part.) | Y | Y | Y | Y (This papers shows that multivariate analyses was not conducted for postnatal care for quantitative part as responses were very low.) | Y | Y | Y | N (This paper separately analysed results for qualitative and quantitative parts and in discussion section interpretation was mainly done based on quantitative results.) | N (This paper only shows the differences in results section but not found any explanation in discussion.) |  | 14 (82.0)  High |
